# Incidence of vestibular schwannoma in Finland, 1990–2017

**DOI:** 10.2340/1651-226X.2024.20352

**Published:** 2024-03-28

**Authors:** Aino Iivanainen, Jani Raitanen, Anssi Auvinen

**Affiliations:** a Faculty of Medicine and Health Technology, Tampere University, Tampere, Finland; b Faculty of Social Sciences, Tampere University, Tampere, Finland; c UKK Institute for Health Promotion Research, Tampere, Finland; d STUK – Radiation and Nuclear Safety Authority, Environmental Surveillance, Vantaa, Finland

**Keywords:** Neuroma, acoustic, cranial nerve neoplasms, incidence, epidemiology, Finland

## Abstract

**Background:**

An increasing trend in incidence of vestibular schwannomas (VS) has been reported, though not consistently, across populations.

**Materials and methods:**

We obtained data from the Finnish Cancer Registry on 1,149 VS cases diagnosed in 1990–2017 with tabular data up to 2022. We calculated age-standardised incidence rates (ASR) overall, by sex, and for 10-year age groups. We analysed time trends using Poisson and joinpoint regression.

**Results:**

The average ASR of VS in Finland during 1990–2017 was 8.6/1,000,000 person-years for women and 7.5/1,000,000 for men. A declining trend was found with an average annual percent change of −1.7% (95% confidence interval [CI]: −2.8%, −0.6%) for women, −2.2% (95% CI: −3.6%, −0.7%) for men, and −1.9% (95% CI: −2.9%, −1.0%) for both sexes combined. The ASR in women was 11.6/1,000,000 person-years in 1990 and it decreased to 8.2/1,000,000 by 2017. Correspondingly, the incidence in men was 7.1/1,000,000 in 1990 and decreased to 5.1/1,000,000 by 2017. Some decline in incidence over time was found in all age groups below 80 years, but the decline (2.3–3.1% per year) was statistically significant only in age groups 40–49, 50–59, and 60–69 years. In the oldest age group (80+ years), the incidence of VS increased by 16% per year. For 2018–2022, the ASR was 7.6/1,000,000 for both sexes combined, with a decline by −1.7% (95% CI: −2.3%, −1.2%) annually for the entire period 1990–2022.

**Conclusion:**

In contrast to the increasing incidence reported in some studies, we found a decreasing trend in VS incidence for both sexes in Finland.

## Background

Vestibular schwannomas (VS), also called acoustic neuromas, are benign tumours that originate from Schwann cells surrounding axons in the vestibular branch of the eighth cranial nerve. Schwannomas constitute 6–8% of all intracranial tumours [1, 2]. Though schwannoma is a slow-growing benign tumour, it can damage surrounding tissues. Characteristic symptoms are hearing loss, tinnitus, and dizziness due to tumour compressing the eighth cranial nerve [2–5]. Most VS cases are sporadic and unilateral. Bilateral cases are closely related to neurofibromatosis type 2, which carries an exceedingly high risk for bilateral VS [2, 5–7]. Other suggested risk factors include allergies, radiation treatment in childhood, noise exposure, and mobile phone use [8,9,10], but the evidence for their role remains inconclusive.

According to several studies, the incidence rates of VS have increased during the past few decades [2, 11–14], though in some studies no clear changes have been reported [15, 16]. Increased incidence rates might be attributable to several reasons. It has been suggested that expanding use of improved diagnostic technologies (mainly magnetic resonance imaging [MRI] and computed tomography [CT]) has improved the detection of VS [2, 5, 12, 13, 17–20], but that these alone would not fully explain the increase in incidence [14]. Almost a quarter of VS diagnoses are incidental [12].

A study with data from the Danish, Swedish, Norwegian, and Finnish cancer registries for 1978–2007 [15] reported that the incidence of VS has decreased slightly in Finland, but the decline was not statistically significant. The same study reported the incidence of VS in Finland as 6.1/1,000,000 person-years among men and 6.9/1,000,000 among women. Unlike in Finland, the incidence of VS increased in Denmark, Norway, and Sweden, though in Sweden the increase was not statistically significant. In an analysis combining the data from all countries, an increasing trend was observed.

The aim of this study was to describe VS incidence rates from 1990 to 2017 in the Finnish population. Furthermore, the aim was to investigate changes over time in the incidence rates by sex and age group.

## Material and methods

The case data were obtained from the Finnish Cancer Registry (FCR) for the study period from 1990 to 2017. The FCR is a nationwide, population-based registry controlled by the Finnish Institute for Health and Welfare. The FCR covers 95% of all cancers in Finland [21]. From 2008, all reported cancers are coded using the ICD-O-3 classification [22]. Previously used ICD-7 [23] codes were replaced by ICD-O-3 codes during the transition. Data on population size by sex, 5-year age group, and calendar year were obtained from Statistics Finland. In addition, head region CT and MRI imaging data during 2008–2017 were obtained from the Finnish Institute for Health and Welfare.

### Variables and exclusion criteria

The FCR provided case-specific information on the sex and age of the patient, as well as the date of diagnosis, primary site, histological type, tumour malignancy, and method of confirmation of the diagnosis. In addition, diagnoses based only on a death certificate or autopsy were flagged. No personal identifiers were obtained.

**Figure 1:**
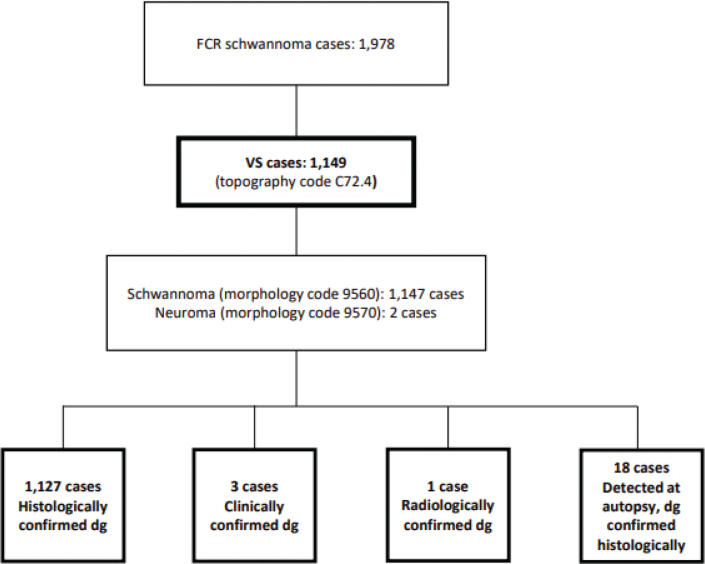
A flow diagram depicting the construction of the material for 1990–2017.

The data covered 1,978 schwannoma cases (Figure 1). Morphology codes 9560 (Schwannoma, ICD-O-3) and 9570 (Neuroma, ICD-O-3) were used for schwannoma. To select vestibular cases, topography code C72.4 (Acoustic nerve) was used. The material contained a total of 1,149 VS cases, of which two were coded as neuromas in morphology and the remaining 1,147 as schwannomas. Out of the 1,149 cases, 1,127 (98%) were histologically confirmed from the primary tumour. Three cases were diagnosed only clinically, and one diagnosis was based on imaging only. Eighteen cases were detected at autopsy and confirmed histologically. In three of these 18 cases, autopsy was the only basis for the diagnosis. None of the cases were based solely on a death certificate.

In an analysis of influence (sensitivity analysis), we also used topography code C71.6 (including cerebellopontine angle) besides C72.4 with 13 additional cases, because VS comprises about 80% of all cerebellopontine angle tumours [24].

Additional data for the most recent years (2018–2022) were obtained in aggregated tabular format (no individual records) for both sexes combined and in 10-year age groups. Due to privacy regulation, frequencies below five were not released and we had to use multiple imputation to assign frequencies of 1–4 cases (see below).

### Statistical analyses

We calculated ASR for VS with 95% confidence intervals (CI) by calendar year, sex, and 10-year age group using the European standard population [25]. We used Poisson regression with Stata statistical software (version 16.0) to estimate rate ratios and their 95% CI. Age-standardised incidence rates were calculated in Excel (version 2008) using direct standardisation with weights from the European standard population.

For analyses of changes in trend over time, joinpoint regression with Joinpoint Regression Program (version 4.8.0.1, U.S. NCI) was used to assess changes in trend and estimate the annual percentage change in ASRs with time segments. When estimating the association of head region imaging with the number of VS cases, we used Poisson regression with Stata.

For the data on case frequencies during 2018–2022, exact numbers of cases were lacking in a total of five cells (out of 30) in the age group – calendar year matrix. We used multiple imputation in Stata (command ‘mi’), generating 10 datasets, where the missing values were imputed (1–4 cases imputed for the analysis) using Poisson regression with the observed population sizes for prediction. Regression analysis was then used for the 10 imputed datasets and imputation results combined using Rubin’s combining rules.

## Results

**Figure 2:**
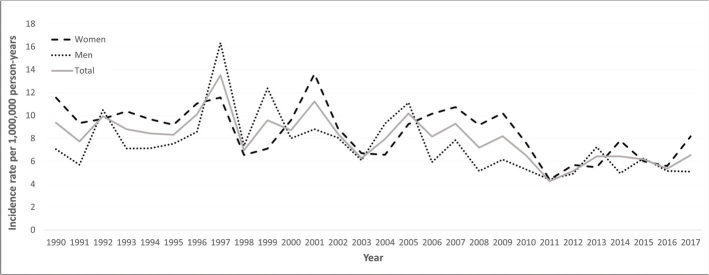
Age-standardised incidence of vestibular schwannoma in Finland by year during 1990–2017.

During 1990–2017, 1,148 VS cases were registered, corresponding to an overall ASR of 8.0/1,000,000 person-years. VSs were more common in women than in men, with ASR 7.5/1,000,000 in men (519 cases) and 8.6/1,000,000 in women (629 cases). (Table 1, Figure 2) The incidence rate ratio (IRR) for women relative to men was 1.15 (95% CI: 1.02–1.29).

**Table 1:** Numbers of cases with crude and age-standardised incidence rates of vestibular schwannoma in Finland during 1990–2017 overall, by sex, age and calendar year.

	*N*	%	Incidence rate per 1,000,000 person-years			
			Crude	95% CI	Age-standardised (Europe)	95% CI
** Overall**	1148	100	7.8	7.3 – 8.3	8.0	7.5 – 8.5
** Sex**						
Men	519	45.2	7.2	6.6 – 7.8	7.5	6.8 – 8.1
Women	629	54.8	8.4	7.7 – 9.0	8.6	7.9 – 9.2
** Age (years)**						
< 40	208	18.1	2.8	2.4 – 3.2	-	-
40–49	220	19.2	10.4	9.0 – 11.8	-	-
50–59	368	32.1	18.6	16.7 – 20.5	-	-
60–69	232	20.2	14.4	12.5 – 16.2	-	-
70–79	107	9.3	9.9	8.1 – 11.8	-	-
80+	13	1.1	2.2	1.0 – 3.4	-	-
** Calendar period**						
1990–1994	205	17.9	8.1	7.0 – 9.2	8.9	7.6 – 10.1
1995–1999	236	20.6	9.2	8.0 – 10.3	9.7	8.5 – 11.0
2000–2004	218	19	8.4	7.3 – 9.5	8.5	7.4 – 9.6
2005–2009	231	20.1	8.7	7.6 – 9.8	8.6	7.5 – 9.7
2010–2014	158	13.8	5.8	4.9 – 6.7	5.8	4.9 – 6.7
2015–2017	100	8.7	6.1	4.9 – 7.2	6.0	4.8 – 7.2

The highest incidence rate was in the age group 50–59 years overall in both sexes (Table 1). Both older and younger age groups had lower rates and the incidence was lowest in the oldest (80 years and older with an incidence rate of 2.2/1,000,000) and youngest ages (below 40 years of age 2.8/1,000,000). In the age groups under 50 years, only minor differences were found between men and women. In the age groups 50–69 years, women had higher incidence rates, but this was reversed after 70 years of age, with higher rates in men.

**Figure 3:**
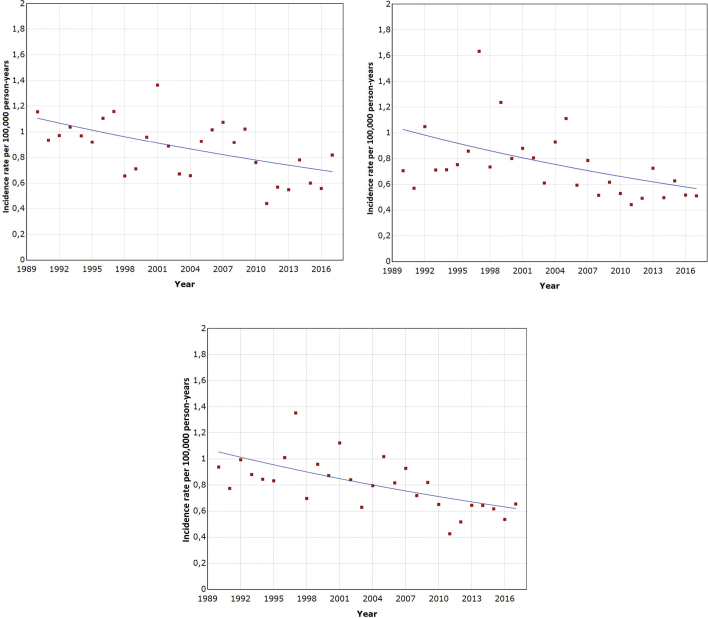
Incidence trend of vestibular schwannoma in Finland for women (A), men (B), and both sexes combined (C) from joinpoint regression.

We found a decreasing trend in the incidence of VS for both genders and in total (Figure 3). The average annual percent change (APC) for men was −2.2% (95% CI: −3.6%, −0.7%), for women −1.7% (95% CI: −2.8%, −0.6%), and total −1.9% (95% CI: −2.9%, −1.0%). Overall, the annual decline in the incidence of VS was −2.0% (IRR: 0.98; 95% CI: 0.97–0.99). Because of the revision of coding at the FCR in 2008, we analysed the incidence trends separately before and after it. From 1990 to 2008, the annual decline was −0.5% (IRR: 0.995; 95% CI: 0.98–1.00) and from 2009 to 2017, it was −1.7% (IRR: 0.98; 95% CI: 0.94–1.03). In the age groups 40–49 and 60–69 years, a statistically significant decline in incidence over time was found. The decline was from 2.3 to 3.1% per year. Incidence decreased non-significantly in age groups 40–49 (−0.6%) and 70–79 years (−1.5%). Only in the age group 80+, a statistically significant increase (16%; 95% CI: 4.2–29.2%) was detected in incidence.

In the joinpoint analysis, no breakpoints in the incidence trend were detected, indicating a constant decline throughout the study period (1990–2017).

In an analysis of influence, 13 additional cases coded as benign tumours of the cerebellopontine angle and one semi-malignant tumour were also included in the material (with a total of 1,162 cases). The results were similar to the main analysis, indicating a decreasing incidence. For men, the mean APC was −2.1% (95% CI: −3.7%, −0.6%) and for women −1.7% (95% CI: −2.8%, −0.6%) with these additional cases included.

An ancillary analysis was conducted incorporating also the years 2018–2022 for both sexes combined and imputation for five age group-calendar year strata with 1–4 cases. It gave APC −7.5% (95% CI: −16.7%, +2.7%) for 2018–2022, and −1.7% (95% CI: −2.3%, −1.2%) for the entire period 1990–2022.

With Poisson regression, we estimated the association of head region CT and MRI imaging with the number of cases of VS during 2008–2018. For both imaging methods separately and together, estimated IRRs for the association between the annual numbers of radiologic examinations and numbers of VS cases were very close to 1, with *p*-values 0.32 or above. Because the reported number of imaging examinations was unrealistically small during the first 2 years (2008 and 2009), we excluded them from further analysis, but the results remained essentially unchanged, with IRR close to 1 and *p*-values above 0.32. The results do not hence indicate the frequency of diagnostic examinations as a clear determinant of VS incidence.

## Discussion

We found a significantly decreasing trend in the incidence of VS by approximately −2% per year, from approximately 10 to 7 per million per year, comparable among men and women. A decreasing trend was also found in most age groups, except for the oldest ages with small numbers of cases and imprecise estimates. An ancillary analysis for the years 2018–2022 (with tabular rather than individual data and imputation of case counts for five cells with frequencies 1–4) confirmed the decreasing trend also during the latest period. This result also indicates that the decline is unlikely to be due to simply incomplete coverage (delayed reporting) during the latest years.

Our results are consistent with an earlier analysis [15] showing a decreasing incidence of VS in Finland up to 2007. The estimated incidence rate in the current study was comparable to the earlier results (approximately 6 per million when standardised to the world population [26] as in the earlier study, instead of the European population used in the current analysis). Also, when we calculated the APC for a period that corresponds as closely as possible to the period of the previous study, we obtained similar results. In the previous study, the decreasing trend was less prominent (APC −0.16 for men and −0.70 for women). The earlier study involved four Nordic countries, with a 20-year study period up to 2007 and approximately 900 VS cases in Finland. Our study period covered 28 years ending in 2017 with roughly 1,150 VS cases. We analysed incidence trends also using joinpoint regression to detect changes in trend, which was not used in the previous study.

In some other countries, increasing trends of VS have been reported [11–14, 17–19, 27–29]. In Danish studies [13, 15, 17, 18, 27–29], incidence increased from about 3–7/1,000,000 in the 1970s to 10–20/1,000,000 after the year 2000. The highest incidence was 34/1,000,000 in 2015 [13]. Most previous studies have not reported ASR. Kleijwegt et al. [29] reported Dutch rates standardised to the European standard population with an overall incidence of 10/1,000,000 person-years in 2001 increasing to 15.5/1,000,000 by 2012. These results are comparable to ours but with a slightly higher incidence. Also, in the Nordic study by Larjavaara et al. [15], rates in Denmark were higher than in Norway, Sweden, and Finland.

In our study, the age group 50–59 years had the highest incidence rates and incidence decreased toward older age groups. Also, other studies have reported the highest incidence rates at ages 55–74 years [12, 16, 19, 29, 30, 31]. Some studies have shown higher incidence rates for women than men and others vice versa, while yet others show no difference. Hence, the reported sex pattern in VS is inconsistent [11, 12, 16, 18, 30, 31]. In our study, women had a significantly higher incidence than men.

In the Unites States of America (US), lower incidence rates than in Denmark have been reported. In the 1990s, the incidence was approximately 6–8/1,000,000 person-years [11] and in the 2000s to 2010s, roughly 11–19/1,000,000 with rates standardised to the US 2000 standard population [16, 30, 32]. Kshettry et al. [16] and Cioffi et al. [30] who reported incidence rates close to 11/1,000,000 had data for the entire US population. Withrow et al. [32] reported higher incidence rates, but they used SEER cancer registry network data covering only approximately 28% of the US population.

In an English study [19], the crude incidence of VS was 11.8/1,000,000 person-years and in Taiwan [31], it was 26.6/1,000,000. In a systematic review examining the incidence of primary brain tumours internationally, the incidence of VS ranged 3–12.7/1,000,000 person-years, although only two studies in the meta-analysis investigated VS and the incidence rates were not age-standardised [33]. The study data were from 1987–2000 and study periods spanned only from one to 5 years. Also, data sources and diagnostic criteria varied between the two studies.

Similar to our results, Kshettry et al. [16] observed a non-significant decreasing trend in the incidence of VS up to 2010, while Cioffi et al. [30] reported an increasing incidence with an APC of 1.64% with later data up to 2016. Withrow et al. [32] showed no changes in incidence overall but found a decrease in the incidence rates of microscopically confirmed cases, while rates of radiologically confirmed cases increased from 2004 to 2017. Marinelli et al. [34] suggested that register-based studies underestimate the incidence of VS in the US. This would be because registers rely on diagnoses from pathology specimens and cancer-related treatment data but can miss benign cases when clinical diagnoses are not confirmed histologically. Marinelli et al. [34] found an increasing incidence trend in a population-based cohort with three to four times higher rates compared with an institutional tumour registry that showed no increase in incidence. Leinonen et al. [21] estimated that the FCR covers 85% of all benign tumours of the central nervous system, suggesting fewer cases are missed and register-based data are more comprehensive in Finland.

Overall, previous studies have frequently reported increasing incidence, but not entirely consistently. A possible explanation for the discrepancy may be that the reported increases do not represent a genuine increase in VS, but artefactual influences such as more active diagnostic efforts, improved access to MRI, and increased incidental findings [2, 5, 12, 13, 17–20]. Also, older age at diagnosis and decreased diagnostic tumour size over time have been reported [2, 5, 12, 13, 27, 28], which is consistent with possible improved detection. Besides the small numbers of cases, increased imaging could also explain the increasing incidence in the oldest age group in our study. However, we found no significant association between head region imaging and the incidence of VS during 2008–2017 overall. Therefore, changes in imaging do not appear to explain the slightly decreasing incidence of VS in Finland.

Several possible explanations related to health care practices have been proposed for the reported differences in VS incidence [35]. Strikingly high rates of incidental findings have been reported in the US Olmsted County, where about third of population has had an MRI of the head by the age 70 years [36]. Marinelli et al. [14] reported that although the frequency of MRI examinations plateaued from 1995 to 2016, the incidence of incidental findings did not. Easy access to MRI has also been suggested in Denmark where comprehensive health care is publicly funded and so is the consultation of an otorhinolaryngologist, and thus access to imaging can be obtained without a referral [13]. In Finland, access to tertiary care is only through referral from primary acre and rates of head imaging (CT and MRI) are low compared to many other countries (though not the UK). This could partly explain the relatively low VS incidence in Finland compared to other countries, and lack of a strong association with frequency of head imaging. No specific exposures, such as loud noise or use of mobile phones [14], have been identified that could account for the changes in incidence. Overall, healthcare practices and accessibility of imaging are more likely to explain the differences in incidence between studies, as well as changes in incidence over time.

Some uncertainties need to be considered in the interpretation of our findings. With the revision of coding at FCR in 2008, ICD-7 codes were converted to the currently used ICD-O-3 codes. This transition improved the accuracy of the registry data after 2008 [37] and therefore we analysed the incidence trends separately before and after the year 2008. However, a similar non-significant decreasing trend was found for both periods with no breakpoints in joinpoint analysis. Also, we were unable to separate symptomatic and asymptomatic cases or sporadic VS versus NF-2 cases, as in hospital-based case series, because bilateral cases could not be identified. The number of cases in our study is comparable to previous studies [11, 17,18,19, 27, 28]. We were not able to obtain individual data for the latest period (2018–2022), but had to use aggregated data instead, with a further limitation that small numbers of cases (<5) were not released. This affected only five cells and we employed multiple imputation methods to predict the values and were able to estimate incidence trends for the five additional years.

Among our strengths, the FCR is a nationwide registry with a high level of completeness due to mandatory reporting and multiple sources of information, including clinical and pathological notifications and annual linkages with nationwide hospital discharge and death certificate databases [21]. Furthermore, we employed modern statistical methods for examining the incidence trends with both Poisson regression and joinpoint regression.

In conclusion, we did not observe any increase in the incidence of VS in Finland during 1990–2017 or up to 2022, but instead a constant, slightly declining trend from around 10 per million to approximately 7 per million.

## Data Availability

The current Finnish privacy regulation is very stringent and does not allow sharing individual data with sensitive information such as health status outside the research group defined in the permission. Therefore, releasing the original data requires a new permission from the relevant authority (THL). Tabulations can be shared if there are no cell frequencies <5.
